# Graduate Training in the Faculty of Nursing at Universidad de Antioquia. A Contribution to Society’s Needs for Care

**DOI:** 10.17533/udea.iee.v43n3e18

**Published:** 2025-11-06

**Authors:** Elvigia María Posada Vera

**Affiliations:** 1 Nurse, PhD. Professor. Faculty of Nursing at Universidad de Antioquia; Medellín, Colombia. Email: emaria.posada@udea.edu.co . Corresponding author. https://orcid.org/0000-0003-3944-8788 Universidad de Antioquia Faculty of Nursing Universidad de Antioquia Medellín Colombia emaria.posada@udea.edu.co

**Keywords:** students, nursing, education, nursing, schools, nursing, education, graduate., estudiantes de enfermería, educación en enfermería, facultades de enfermería, educación de postgrado., estudantes de enfermagem, educação em enfermagem, escolas de enfermagem, educação de pós-graduação.

## Abstract

Creation of graduate studies as academic programs in the Faculty of Nursing at Universidad de Antioquia began during the 1980s as a result of the generation of knowledge product of profound academic discussions in research and disciplinary areas. The Master’s, specialization, and PhD programs have generated an important academic production that helps make the Faculty visible in the world. These programs are permanently subjected to self-evaluation processes, which lead to improvement plans aimed at raising their quality in response to scientific progress, social needs, and health and education policies within the framework of graduate training for nursing professionals, which guarantees the social pertinence of their formation. Currently, the Faculty’s graduate programs are pertinent in their purpose of training professionals capable of confronting society’s heterogeneity and proposing solution alternatives according to the new challenges from the context to live with quality and dignity. This text seeks to show the historical development of graduate training in the Faculty of Nursing at Universidad de Antioquia in its 75 years of existence and its contribution to care and development of nursing in our society.

## Resumen

La creación de posgrados como programas académicos de la Facultad de Enfermería de la Universidad de Antioquia se inició en la década de los 80 como resultado de la generación de conocimiento producto de profundas discusiones académicas en las áreas investigativa y disciplinar. Los programas de maestrías, especializaciones y doctorado han generado una importante producción académica que ayuda a la visibilización de la Facultad ante el mundo. Estos programas están sometidos permanentemente a procesos de autoevaluación, que conllevan a planes de mejoramiento orientados a elevar su calidad en respuesta a los avances científicos, a las necesidades sociales y a las políticas de salud y de educación en el marco de la formación posgraduada de profesionales de enfermería, lo que garantiza la pertinencia social de su formación. En la actualidad, los programas de posgrados de la Facultad son pertinentes en su propósito de formar profesionales capaces de enfrentar la heterogeneidad de la sociedad y proponer alternativas de solución de acuerdo a los nuevos desafíos del contexto para vivir con calidad y dignidad. Este texto pretende mostrar el desarrollo histórico de la formación posgradual en la Facultad de Enfermería de la Universidad de Antioquia en sus 75 años de existencia y su contribución al cuidado y desarrollo de la enfermería en nuestra sociedad.

## Resumo

A criação de programas de pós-graduação como programas acadêmicos na Faculdade de Enfermagem da Universidade de Antioquia teve início na década de 1980, como resultado da geração de conhecimento resultante de discussões acadêmicas aprofundadas nas áreas de pesquisa e disciplinas. Os programas de mestrado, especialização e doutorado geraram uma produção acadêmica significativa que contribui para a visibilidade da Faculdade em todo o mundo. Esses programas são submetidos permanentemente a processos de autoavaliação, que levam a planos de melhoria que visam elevar sua qualidade em resposta aos avanços científicos, às necessidades sociais e às políticas de saúde e educação no âmbito da formação de pós-graduação para profissionais de enfermagem, garantindo assim a relevância social de sua formação. Atualmente, os programas de pós-graduação da Faculdade são relevantes em seu propósito de formar profissionais capazes de enfrentar a heterogeneidade da sociedade e propor soluções alternativas de acordo com os novos desafios do contexto, a fim de viver com qualidade e dignidade. Este texto tem como objetivo mostrar o desenvolvimento histórico da formação de pós-graduação na Faculdade de Enfermagem da Universidade de Antioquia ao longo de seus 75 anos de existência e sua contribuição para o cuidado e o desenvolvimento da enfermagem em nossa sociedade.

## Introduction

Given that nursing is a social and practical discipline, its practice and development are influenced by the complex phenomena of an accelerated context that impacts morbidity and mortality profiles and, to that extent, requires renewal of care plans to satisfy new social needs, therefore, highly qualified personnel is needed to lead relevant and timely processes in response to the care needs of each environment. An effective way of facing these challenges is the creation of graduate programs that stimulate the scientific-technical development of nursing in the continuous search to improve the quality of care and its consolidation as science and discipline. The creation of graduate studies as training programs in the Faculty of Nursing at Universidad de Antioquia required profound academic discussions and excellent research training of its professors, evidenced in the formation of groups and lines of research with a high scientific production as a necessary input for their development. These graduate programs focus on four specializations, two master’s, one disciplinary PhD and an interdisciplinary PhD to successfully meet the social, professional, and disciplinary needs of individuals, families and groups.

### Beginnings of a Dream: Advanced Training

The Nursing program at Universidad de Antioquia, with 75 years dedicated to the formation of professionals, has had important development that have led it along paths of professional and disciplinary growth around care, its object of study. By the year of its founding, there were already inter-American organizations in the country, like the Pan-American Health Organization and entities such as the Rockefeller and the WK Kellogg foundations, which supported the creation of nursing schools with funding and training, while also granting scholarships to train their professors in the areas of health and education, which became effective in Universidad de Antioquia in the late 1950s.^1^

This opportunity favored the qualification of professors, and by 1963 there were three specialists in medical-surgical nursing and two in public health. This was potentiated in the 1960s and 1970s with graduate training of several professors in national and international universities and with internships in numerous training centers. In 1997, professor Mabel Restrepo, in her book “History of the Faculty of Nursing at Universidad de Antioquia”, considered that as the School was permeated with more advanced knowledge and other visions of nursing in terms of profession and discipline, its academic growth was on the rise, which strengthened the capacities of the professors and their active participation in interdisciplinary research groups that later became the niches that supported the creation of the Research Center and the first graduate programs in the already renowned Faculty of Nursing. 

By the 1980s, development in research, the presence in the Faculty of a greater number of professors with specialization and master’s degrees, the needs of the context, and guidance by ACOFAEN for the time were reasons that motivated the creation of graduate studies. Parallel to the interest generated in creating the journal and the research center, another project that gained relevance was the creation of several specialties in the clinical areas of medical-surgical and rehabilitation, and another in community health. Consequently, the Faculty Council, convinced of the need and importance of supporting graduate formation, created the graduate studies policy through Agreement 003 of 23 January 1990, which favors both academically and administratively the execution of these projects. Despite having achieved approval from the curricular committee, the first project continued being debated until considering of greater social pertinence a specialization in nursing care of patients in critical state, given the increase in intensive care units and specialized operating rooms in the country, which favored the future employment of graduates and generated greater impact on care.^2^

### Academic-administrative structure of the graduate programs

Once the graduate policy was approved, there was greater interest in consolidating projects in relation to the proposed programs. One of these began in 1992, which required administrative and academic support that guided and backed the development of the programs. This is how, in the organizational restructuring of the Faculty, given in 1994 by Superior Agreement 005 of May 19, the Department of Extension and Graduate Studies was created. Thereafter, the University responded to its growth in the offer of graduate programs, considered fundamental to achieve academic excellence, by creating the University Graduate System through Superior Agreement 058 of 4 December 1995, which regulated the area’s academic and administrative activities, with its autonomy, decentralization, deconcentration, and coordination, as important cores. This agreement also defined what criteria and procedures must be brought conducted in the university to create and offer these programs. Said norm was modified by Superior Agreement 306 of December 2005 that established the system’s functions, making the Graduate Program Committees, Graduate Committees of each department, Faculty, School or Institute Councils, Graduate Area Committees (exact and natural sciences - health sciences - social or human sciences), the Central Graduate Committee, the Academic Council, and the University Higher Council responsible for their compliance, in that order. Moreover, graduate training levels were recognized, such as specializations, master’s and PhD degrees, specifying the scope of each of them. 

By 1998, the University’s Graduate Department was created by Superior Agreement 149 of August 10, to ​​ensure the quality of its programs, from the interdisciplinarity, heterogeneity, and efficiency of its processes in direct relationship with research. The existence of this link favored the organization, development, and consolidation of groups and lines of research. In keeping with the foregoing, in 2025 the Faculty has five groups classified by COLCIENCIAS: *Nursing Practice in the Social Context* (category A1), *Social Policies and Health Services* (category A), *Women’s Health, Health Promotion, and Emergencies and Disasters* (category C), with 23 lines of research. These groups and lines of research fostered key areas of knowledge, where students and professors could conduct socially pertinent research that contribute to the discipline and respond to solving specific problems derived from the practice. Through these lines, communication is also established with peers from other national and international universities to evaluate research work, thus increasing the reliability and quality of the knowledge generated in said work and in turn nourishing the interdisciplinary perspective of the programs.

### Normative platform

Within the framework of the University System, a broad regulatory platform was established that regulates all the academic and administrative processes of graduate studies in Universidad de Antioquia, as a compass for action on which these processes are based in the Faculty of Nursing, with its own regulations when authorized by the central structure. In broader terms, the general student regulations for graduate programs (Superior Agreement 432 of 25 November 2014) and a specific one for each unit are mentioned, endorsed in Nursing by Agreement 097 of 26 September 2018. Currently being updated, both regulations are supported by the graduate student affairs committee, whose function is to review and resolve students' academic requests. The compulsory nature of a second language is regulated as a central aspect to support internationalization of the curriculum, international mobility of students through strategic alliances that favor agreements, internships, exchanges, scholarship management and scientific and cultural collaboration events, as well as participation in international knowledge networks, which permits access to new sources of information and increased scientific production to improve the quality of the programs. All this was supported by the University’s Interinstitutional Nursing Cooperation Committee and the International Relations Department. 

### Quality conditions

Adherence to and ongoing evaluation of the quality conditions established in Decree 1330 of 25 July 2019 accounts for the social commitment and responsibility of the Faculty of Nursing with the academic excellence of its formative processes. From the Graduate Department, work is underway regarding this objective, through self-assessment and ongoing evaluation processes with participation from internal and external stakeholders that results in an improvement plan, in search of obtaining or renewing the qualified registrations of each of the programs and the accreditation of those that meet said requirements, such as the Master’s in Collective Health, already accredited, and the Master’s and PhD in Nursing in process of being accredited. 

### Graduate programs in the Faculty of Nursing: a contribution to the development of the profession and to socially pertinent care

1. **Specialization in Nursing Rehabilitation**

This was one of the projects proposed towards the late 1980s, becoming a reality through ICFES Resolution 082 of 4 June 1992 designed for three face-to-face semesters with a preventive focus, with exclusive dedication and practical theory. This program began in February 1993 and had 27 graduates distributed into three non-continuous cohorts, granting the title of Specialist in Nursing Rehabilitation. It closed in 2000, at the end of the third cohort, given that the purpose of its creation to cover the need for professionals in the field had been fulfilled; the limited labor recognition by employing institutions and availability of the physical therapy program in other educational institutions were also considered, which risked the job offer for graduates. 

2. **Master’s in Collective Health. Interdisciplinary project with validity in its social relevance.**

According to the Master Document of the Collective Health Program, the idea of ​​specialization in the community area, which was conceived in the 1980s, was nourished by the discourse and theories of the social medicine movement in Latin America, which permeated the Faculty with greater strength in the early 1990s, posing a critique of the neoliberal model and a biomedical and positivist perspective focused on disease and epidemiological problems unresolved by healthcare systems.^1^ These reflections identified challenges that shifted research toward other more qualitative and comprehensive axes, increasing scientific production that was strengthened by the view of national and international experts, and contributing to shaping a serious, solid, and flexible program that was shaped after a profound interdisciplinary discussion in the workshop “Social sciences in graduate programs in social medicine in Latin America and in the Master’s in Collective Health”. This master’s was approved via academic Agreement 166 of 26 November 1991, and created by Superior Agreement 197 of 11 December of the same year and, finally, approved by the Colombian Institute for the Evaluation of Education through agreement 177 of August 13, 1992. It began its first cohort in 1994 with a study plan focused on understanding health articulated to social processes and a research emphasis from a critical perspective that questions and seeks to impact conditions and quality of life of human groups.^1^

Constant efforts to continually improve the program’s quality allowed it to receive the “Luis López de Mesa Order on Education and Public Faith” from the National Ministry of Education, through Resolution 10567 of December 2011. In 2012, it extended to the Urabá region in Antioquia, with Qualified Registration granted for seven years, by Resolution 10407 of November 26, 2010, where only one cohort with three graduates was offered. Its quality has been certified and accredited by the National Ministry of Education under strict internal and external evaluation processes; currently, with qualified registration number 008737 of 30 April 2025 for seven years, extended to the Urabá region, and accredited until 2027, through Resolution 018122 of 27 September 2021. In 2025, the master’s added 31 years of being offered under the guidelines established for graduate formation in the University, maintaining its initial purpose of training professionals from different disciplines, under the analytical articulation of social sciences and health, recognizing the political and economic influence exerted on the practices of the biomedical model and the need for intersectoral effort in meeting this sector’s needs. Currently, the Master’s in Collective Health has 100 graduates in Medellín and 3 in Urabá, who are professionals from the social areas of health, which accounts for understanding the necessary interdisciplinary approach to complex problems, such as caring for human beings. 

3. **Specializations in Adult Care and in Care of Children in Critical Health Conditions**

The experience acquired, enhancement of knowledge, greater research production, and a sounder academic-administrative structure were the elements that drove the creation of the Specialization in Nursing in Care of Adults in Critical Health Conditions, whose project was being worked on since the early 1990s in response to the needs of a complex health panorama with high mortality and morbidity indices that required urgent and specialized care in high-technology units, such as intensive care, which required nursing professionals with greater training, capable of responding to the complexity of the needs of the subject of care and the management of the clinical-administrative processes that will support such care; a challenge assumed by the Faculty of Nursing by creating two specializations - one in Care for Adults in Critical Health Conditions (Resolution 001050 of 1 February 2022), and another in Care of Children in Critical Health Conditions (Resolution 013239 of 8 July 2022 from the Ministry of Education), being the first higher education institution in Antioquia to prepare specialists in this area. 

Both specializations were approved by Academic Agreement 109 of 15 July 1997, with a duration of three semesters, granting the title of Specialist in Nursing in Care of Adults in Critical Health Conditions or Specialist in Nursing in Care of Children in Critical Health Conditions. The program’s academic axes focused on the care of individuals in critical health conditions, as an object of study, research through structuring monographic works, management of Intensive Care Units, and practice in Intensive Care Units and Special Care Units or Intermediate Care, in addition to two elective courses to delve into the care of patients with critical health disorders of neurological and cardiovascular origin. 

Nowadays, the pertinence of these specializations is still valid given the characteristics of the health context, which evidences, among other things, the transition from the epidemiological profile with prevalence of rare diseases, trauma, increased cancer and decompensated chronic diseases; along with transplants and interventional radiology therapies, which lead to an increased demand for care in critical care units with greater scientific and technological progress, requiring greater training for healthcare staff. Thus, the formative components are aligned with five axes of the Educational Project of the Graduate Department Program: care, disciplinary foundation, bioethics, management and research, considered key elements in humane and quality care of patients in critical care. The aim is for specialist nurses to acquire skills to work in ICUs, coronary and transplant units, third-level emergency services, interventional radiology, hemodynamics and electrophysiology, among others, by integrating knowledge in reflective and coherent manner, in concordance with the demands of patients, the structure of services and institutional policies, and the Colombian healthcare system. Both programs have obtained qualified registration for three consecutive times, the last of these in 2022, with an extension of the opening to the eastern campus of Universidad de Antioquia, both for seven years. 

Bearing in mind the guidelines by the National Ministry of Education, and the University’s projections regarding graduate programs, Both specializations diminished their duration to two semesters, in line with other programs in the country, delivering trained professionals in less time with quality training at lower costs.^3^ In 2025, the Nursing specialization in Care for Adults in Critical Health Conditions completed cohort 16 with 179 graduates and the specialization of Child Critical Care has 14 cohorts with 99 graduates.

4. **Specialization in cancer patient care**

The master document of the Specialization in Oncological Nursing Program indicates that the interest by the Faculty of Nursing in training professionals capable of responding to the health needs of the environment and the high rates of morbidity and mortality due to pathologies, like cancer, justified in 1996 this specialization’s creation, which was carried out in association with Pontificia Universidad Javeriana. The idea was to train clinical professionals with a comprehensive approach to preventive, curative and rehabilitative care for cancer patients and their families, also seeking the development of research in this field. In 1999, eleven specialists graduated of which four were professors from the Faculty; this program only carried out one cohort.

Given that the need for these specialists was on the rise, evidenced on the increased global incidence of this disease,^4^ seen as one of the diseases with the greatest human and social cost, the Faculty again considers the creation of the program to train competent specialists to provide specialized care to those suffering from cancer through a reflective practice inspired by the different conceptualizations that exist from the discipline for care and which evidence needs for applied research in the area. This program was created by Academic Agreement 387 of 3 February 2011 as Nursing specialization in Care of Cancer Patients and their Families, initiating the first cohort in 2013, based on the postulates of the nursing metaparadigm: care, person, context, nursing and health-disease process, considering epistemological, conceptual, and methodological elements of the discipline to support practice. 

In February 2021, during the second renovation process of the qualified registration, the National Ministry of Education recommended adjusting the program’s name to a more precise denomination, in concordance with that generally accepted in health specialties, modifying it for Specialization in Oncological Nursing because it is a nationally and internationally recognized denomination. Thus, through Academic Agreement 572 of 25 February 2021, the program’s name change was approved and then endorsed by the National Ministry of Education through Resolution 017583 of 16 September 2021, extending the qualified registry for seven more years, favoring the qualification of professionals and consolidation of the program in the academic and social setting by being the only one offered in the Andean region and throughout north-central Colombia. The study plan for this new version was developed through nuclei focused on oncology nursing, research, and management, promoting critical reflection on care experiences in professional practice, which demonstrate the need for research. Until 2025, eight cohorts have been carried out, seven of them completed with 94 graduates.

5. **Specialization in Maternal Perinatal Nursing**

The Faculty’s Maternal Perinatal Nursing Specialization emerges from the national need for comprehensive, safe, differential, and inclusive care for the maternal perinatal population to diminish morbidity and mortality indicators in said population. In Medellín, gynecology and obstetrics services in health institutions do not have nursing professionals specialized in the area, and those practicing there receive empirical training from professional peers who have the skills, but have no theoretical and practical training from a formal specialization program that certifies their knowledge. 

The program is also offered, in coherence with the requirements of Resolution 3280 of 2018, expressed in the Comprehensive Maternal Perinatal Care Route, which empowers recognized and trained nursing professionals to be leaders in the direct care of low-risk births and continuous care of women and newborns from preconception to postpartum, as a public health commitment to improve the quality of life and health of mothers and children. Its creation, as the only one in the region, contributes to improving the health situation of this population, facilitating access to advanced training for nurses from other areas of the department and the country, supported by a disciplinary and professional approach in the maternal perinatal area. The program is based on concepts and theories of the nursing discipline and on postulates from the biological sciences that allow understanding the physiology of the reproductive process and its alterations. This specialization was created through Academic Agreement 590 of 25 February 2022, endorsed by Ministry of Education Resolution 5526 of 5 April 2023. In 2025, with a cohort of six graduates, it is about to open a second cohort. 

6. Master’s in Nursing

By the 1990s, the arrival of the first female nursing PhDs gave rise to the disciplinary discourse in the country, which was disseminated through academic settings, such as colloquia, seminars, nursing congresses and internships; this, added to the arrival to the Faculty of Nursing of experts in disciplinary concepts modified the mentalities of its professors, who promoted a movement of reflection on disciplinary conceptualization and strengthened research processes on the subject. Thus, epistemological concepts were enhanced, together with the adoption of nursing vocabulary and disciplinary practices, such as the application and use of the Nursing Care Plan and its models and theories. Consistently, publications focused on the disciplinary topic began to increase. Works on this topic favored the promotion and consolidation of disciplinary knowledge in the Faculty, showing a promising perspective for the creation of the master’s degree in nursing,^1^ which was carried out through Academic Agreement 190 of 2001 to deepen the discipline’s scientific knowledge and research to solve specific problems of the practice, thereby, contributing to the profession’s development in the department of Antioquia and the region. The cohort in 2005 had nine graduates, mostly professors from the Faculty, which favored greater positioning of the disciplinary discourse in this academic unit and promoted large projects, such as the subsequent creation of the PhD in nursing.

As the first program of this level in the department, it prioritized in its formation four curricular axes: care, research, discipline, and the profession, supported on other knowledge, such as social and human sciences, ethics, and management of health services. This has allowed graduates to direct their practice towards solving care problems in the context of individuals, families, and human groups within the framework of knowledge of interest to nursing, strengthening the discipline and the transformation and innovation of its practice in care, research, education, consulting and the exercise of citizenship from the field of nursing. 

Within the framework of the Graduate Policy at Universidad de Antioquia, the master’s programs may be offered from two perspectives: in-depth, to delve into an area of ​​knowledge from specific situations to solve problems of disciplinary, interdisciplinary, or professional nature; and research, aimed at developing skills in research processes toward new knowledge. The same program can have these two approaches, with the differentiating elements being the type of research carried out, the credits and the academic activities developed by the students. Hence, The Faculty of Nursing, taking advantage of the qualifications and academic production of its research groups, the knowledge and expertise of its teaching staff and the strengthening of the teaching-service relationship with good practice scenarios, proposes a curriculum for the program with both modalities, approved through Faculty Council Agreement 046 of 2013, and Ministry of Education Resolution 1268 of 12 February 2013. With this new option, students have the possibility of qualifying their transformative and innovative practice by applying the scientific method in the area of ​​emphasis, an important differentiating feature as it is a possibility few programs in the country offer. 

The master’s degrees in depth were offered according to the following phenomena of interest: care for people with chronic health problems, women’s health care, child and adolescent care, care for people in critical health conditions, management, administration and management of care, mental health care in populations, ethics and bioethics of care, care for people with wounds and ostomies, care during the aging process and old age, care for people with cancer. These lines were offered according to needs detected in the program’s self-assessment processes. Guided, observation and field visits were specific teaching modalities for this modality. 

The in-depth line was offered from 2018 to 2021, since in the process of renewing the qualified registration, the National Ministry of Education required independent qualified registrations for each of the modalities, giving real importance in this case to the teaching-service relationship, with a sufficient and effective offer of practice scenarios, sufficiency and availability of expert professors in each of the lines offered by cohort and a sufficiently solid administrative structure to support the program. Considering the time available for the process and the procedures to obtain practice scenarios, it was decided to continue with the research modality, with a future projection to reopen the in-depth modality. 

Two emphasis courses were held: the first on caring for patients in critical health conditions, from which two students graduated; and the second on caring for patients with wounds and ostomies, from which four students graduated. The line of research is ongoing, with eight cohorts developed, seven of which completed with 47 graduates, and an underway cohort with eight students registered. The master's program is currently undergoing high-quality accreditation.

7. **Nursing Doctorate**

Between 1997 and 2001, the Faculty worked on a joint doctoral proposal with five universities: Universidad de Antioquia, Pontificia Universidad Javeriana, Universidad Nacional de Colombia, Universidad Pedagógica y Tecnológica de Colombia, and Universidad del Valle. This proposal was supported by the Association of Schools and Faculties of Nursing and advised by foreign universities, such as the University of California in San Francisco, for the joint design of a PhD degree in Nursing. Due to differences in the academic and administrative autonomy of the participating universities *vis-à-vis* the National PhD Commission, Universidad Nacional with this autonomy closed its internal process and withdrew from the consortium. Therefore, the project under this modality was suspended and halted, and the agreement was canceled by the Rector's Office and the Legal Advisory Office at Universidad de Antioquia.

The autonomy of Universidad Nacional de Colombia in approving the program ended with the Interuniversity Agreement signed for this purpose, and it approved its own PhD program. The other faculties decided to continue their work independently.^1^ This experience enabled participation by professors from the Faculty in multiple national and international training settings regarding the disciplinary topics, debates, and internships in different universities around the world that already had a PhD degree in nursing, increasing their knowledge and qualifications to resume the PhD proposal at the Faculty, added to the conceptual and methodological maturity that had already been achieved in terms of discipline by the first decade of 2000 thanks to the development of the master's degrees in nursing and Collective Health and the growth of the groups and lines of research.

This is how, through Academic Agreement 350 of 7 May 2009, the PhD program in Nursing at Universidad de Antioquia was created, seeking to align itself with the possibilities and needs to develop the profession in the local context and with the trends of professional and research development in the international context, seeking to solve social problems through the generation and empirical verification of theories and application of scientific progress in the practice of caring for individuals and groups.^1^ The program’s first cohort started in 2010 with a study plan that favors the qualification of Nursing professionals for the development of knowledge in autonomous research exercise, with a high humanistic, ethical and high-quality level to deepen in the epistemological aspects of nursing care and the theories and models of the area. It took as central axes care, health, people, and the complex relationships among such categories, as well as the dynamic processes that develop inter-articulated in a complex health context, seeking to strengthen the discipline and the practice with academic, social, and scientific pertinence. By 2025 the program has carried out six cohorts with 25 graduates, a cohort underway with two students registered. 

8. **Education and Health Doctorate: A commitment to transdisciplinarity and cooperation among academic units for scientific training at the University**

The relationship between education and health, as complex phenomenon, must be studied in depth as it is one of the central axes in the implementation of public health policies in the country. Due to this, the demand for training in this field must be addressed from a transdisciplinary perspective within the framework of a PhD program, given the theoretical support required by its practice, which must be resolved through high-level academic research development. To meet this need, Universidad de Antioquia created the PhD in Education and Health, approved through Academic Agreement 614 of 17 April 2024 and Resolution 18498 of 21 October 2024 from the National Ministry of Education. It is the first in this field in Latin America, with great relevance given the limited research development in the line of study proposed.

Cooperative and collaborative inter-professional work facilitates academic work in terms of research and appropriation of knowledge, which strengthens the students’ quality formation with rationality of resources, achieving expansion of research and training development within the university, with a broader social, ethical and political impact. In this sense, the PhD program becomes an opportunity to promote human formation and transformation, from a transdisciplinary, intersectional, and intersectoral perspective that articulates, integrates and recontextualizes the fields of education and health.^5^ This is a proposal for inter-academic unit cooperation, with the participation of the Faculty of Nursing, School of Microbiology, School of Nutrition and Dietetics, Faculty of Agricultural Sciences, Faculty of Medicine, National Faculty of Public Health, University Institute of Physical Education and Sport, and Faculty of Education. Each of them from their specific knowledge, with representation of highly qualified professors and support from groups and lines of research, such as that of social policies and health services and health promotion for nursing, with their respective lines of research, education as a social practice and health education. In all, it will have 10 research groups and 11 lines of research, belonging to the academic units involved.^5^ It is a six-semester PhD program, in face-to-face modality with support from ICTs, aimed at professionals with experience in the health system or in the educational sector. Administratively, it is represented by the National Faculty of Public Health, with academic support and teaching management from all associated units. It began its second cohort during the second semester of 2025 with 15 students registered.

### Academic management of graduate programs

Direct management of the graduate programs is the responsibility of each program’s coordinator, in support of the regulations set forth and the guidelines by the department’s head. From the academic point of view, there is support from the Curriculum Committee, which guides the formulation, development, and modifications of the study plans, which are revised at the beginning of each cohort, to integrate the new knowledge, assuring articulation among the disciplinary, research, practical, philosophical, and ethical components, and - thus - the continuity of its social pertinence. The new demands of educational policy and the administrative and academic needs of the moment are also considered. In this sense, methodologies that integrate information and communication technologies into the teaching-learning process have been implemented, besides strengthening the flexibility of the curriculum by offering elective courses per program and internships that seek to enhance specific formation areas according to students' needs and interests, with a focus on their area of ​​performance. 

### Challenges

In the Faculty of Nursing at Universidad de Antioquia, graduate training is conceived as one of the pillars for social transformation and the search for better levels of wellbeing in the population. The complexity and dynamism of the overall context, the accelerated technological development, and rapid generation of knowledge - characteristics of the current world - require highly qualified personnel in the scientific field, capable of leading these processes and correctly responding to the population’s interests, needs, and problems, which requires mastery of the disciplinary theories and those specific to each area of the technical and research processes. 

Graduate programs are a source of high scientific production whose dissemination through different academic settings makes the Faculty and the University visible and established in the academic world. All this drives its own development and - in turn - generates new research alternatives, which demands continuity in the formation of researchers and enhancement of technical skills in the specific fields required by a society in permanent movement. Considering the current speed in scientific progress, the transitory nature of the contents and approach of graduate programs must be recognized; so, to sustain their social relevance, considerable academic and administrative flexibility is required, which supports the prompt adaptation of their training proposals to the trends of new knowledge. 

The scientific consolidation and visibility that graduate programs give to the University and Faculty also generate internal challenges. It is fitting to consider the national and international demand for the Faculty’s graduate programs, and its in-person modality that makes it difficult for students from other regions of the country and the world to enter, so a challenge is to offer them in bimodal manner, availing of the possibility offered by ICTs of simultaneous connection in real time or asynchronously, without geographical barriers, according to the characteristics of potential students and the sustainability of the programs in the university. 

It is also necessary to strengthen interdisciplinary research with other groups and research lines in the university that allows the use and cooperation to rationalize human, technical, and scientific resources and enhance the generation of knowledge from a broader and more productive perspective. Lastly, the need is recognized to establish a more effective relationship between the graduate and the undergraduate programs, so that the latter is permeated by the theoretical and methodological progress achieved in the first of these. In the discussions, debates, and defense of degree projects, faculty and undergraduate students should be present as a way to promote new projects, stimulate advanced training in them, and more strongly adopt disciplinary and professional issues in the Faculty. 
